# The prevalence of the *ABCB1-1Δ* variant in a clinical veterinary setting: The risk of not genotyping

**DOI:** 10.1371/journal.pone.0273706

**Published:** 2022-08-29

**Authors:** Evy Beckers, Iris Casselman, Emma Soudant, Sylvie Daminet, Dominique Paepe, Luc Peelman, Bart J. G. Broeckx

**Affiliations:** 1 Department of Veterinary and Biosciences, Faculty of Veterinary Medicine, Ghent University, Merelbeke, Belgium; 2 Small Animal Department, Faculty of Veterinary Medicine, Ghent University, Merelbeke, Belgium; Colorado State University, UNITED STATES

## Abstract

Multidrug sensitivity is an autosomal recessive disorder in dogs caused by a 4-bp deletion in the *ABCB1* gene, often referred to as the *ABCB1-1Δ* variant. This disease has a high prevalence in some breeds and causes adverse reactions to certain drugs when given in normal doses. Though most dogs known to be at risk are of the collie lineage or were traced back to it, the variant has also been described in several seemingly unrelated breeds. It is generally advised to genotype dogs at risk before treating them. However, there seems to be a discrepancy between the advice and current veterinary practices, as a recent study in Belgium and the Netherlands showed that most veterinarians never order a DNA test. To assess the possible risk of not testing for multidrug sensitivity in a clinical setting, the *ABCB1-1Δ* variant allele frequency was established in a sample of 286 dogs from a veterinary clinic. This frequency was compared to the allelic frequency in 599 samples specifically sent for genetic testing. While the allelic frequency in the sample for genetic testing was high (21.6%) and in line with the general reports, the allelic frequency in the clinical setting was low (0.2%), demonstrating an enormous difference between laboratory and clinical frequencies. Because of the low frequency of the disease-causing variant in the general clinical population, the risk of encountering a dog displaying multidrug sensitivity despite not genotyping seems to be low. As the variant was only found in an at-risk breed, the current recommendation of routinely genotyping at-risk breeds before treatment seems justified.

## Introduction

Ivermectin sensitivity in dogs, especially in collies, has been reported soon after the introduction of the antiparasitic drug in the early 1980s [1, 2]. The disease-causing variant was identified in 2001 as a 4-bp deletion (*NM_001003215*.*2*: *c*.*230_233del*) in the *ABCB1* gene (previously *MDR1* gene), commonly known as the *ABCB1-1Δ* variant [3]. This deletion causes a frameshift, resulting in a premature termination codon and a truncated, non-functional P-glycoprotein.

The P-glycoprotein belongs to the ATP-binding cassette superfamily and was first described in tumor cells resistant to anticancer agents as a result of *ABCB1* overexpression. This transmembrane protein functions as an ATP-dependent efflux transporter, pumping drugs out of cells. Amongst others, it limits drug penetration into sensitive tissues like the brain, where the P-glycoprotein is part of the blood-brain barrier [4]. As ivermectin targets gamma-amino-butyric acid (GABA)-gated chloride channels, which in mammals are restricted to the central nervous system, P-glycoproteins are crucial in the protection against possibly lethal neurological side-effects of this drug [3]. Dogs homozygous for the *ABCB1-1Δ* variant and thus without functional P-glycoproteins exhibit neurotoxic symptoms such as ataxia, lethargy, coma, tremors, seizures, mydriasis, and blindness at ivermectin doses that are not toxic for wildtype dogs.

Ivermectin sensitivity was later renamed multidrug sensitivity since more drugs have been identified to cause neurotoxic symptoms or other adverse reactions in *ABCB1-1Δ* homozygous animals over the years, like various macrocyclic lactones [5], loperamide [6], acepromazine [7], and several chemotherapeutic agents [8]. The disorder is generally deemed to show an autosomal recessive pattern of inheritance. Intoxications are often more severe and long-lasting in homozygous dogs [9], but it does have to be mentioned that heterozygotes also have been reported to display increased susceptibility to some drugs at certain doses [10]. For example, homozygotes or heterozygotes are more likely to develop hematologic toxicity after treatment with vincristine [11], excessive immunosuppression has been described in a heterozygous carrier after cyclosporin treatment [12], and an intermediate phenotype with milder neurotoxic symptoms concerning ivermectin sensitivity has been reported as well, presumably in heterozygous dogs [9, 10]. However, it is unclear if the intermediate phenotype described for ivermectin is truly related to a heterozygous genotype, as subchronic neurotoxicity can also occur in “normal” dogs (i.e. dogs homozygous for the wildtype allele) under high-dose treatment with macrocyclic lactones [13].

The *ABCB1-1Δ* variant is not only important regarding the use of multiple drugs, but the same causal variant was also discovered in multiple collie-like breeds such as the (miniature) Australian shepherd dog, English shepherd, McNab, Old English sheepdog, border collie, Shetland sheepdog, Wäller, and bearded collie (only one dog was genotyped as heterozygous in this last breed) [14–17]. Moreover, the variant appears in seemingly unrelated breeds like the longhaired whippet, silken Windhound [15], white Swiss shepherd [18], German shepherd [19], cocker spaniel, labradoodle [20], and crossbreeds [21, 22]. Many population studies on the prevalence of the *ABCB1-1Δ* variant have been conducted all over the world [15–17,19–28] and high variant allele frequencies are mainly seen in the collie, (miniature) Australian shepherd, Shetland sheepdog, and longhaired whippet. Therefore, it is generally advised to genetically test these predisposed breeds for multidrug sensitivity before medicating them with any of the risk drugs [5, 27]. However, a recent cross-sectional study in Belgium and the Netherlands on the use of genetic tests in the daily veterinary practice showed that 55% of university-employed veterinarians never request a DNA test [29]. Consequently, these veterinarians also never request a test for multidrug sensitivity, while veterinarians typically administer many types of drugs, including those causing adverse reactions in *ABCB1-1Δ* homozygous dogs. While many population studies specifically target breeds (possibly) at risk, no population studies targeting the general clinical population have been conducted to date. Therefore, this study aimed to quantify the probability of being presented with a multidrug-sensitive patient and the risk this brings for veterinarians who do not test.

## Materials and methods

### Sample collection and DNA extraction

Residual EDTA blood samples of dogs were collected at random from the laboratory of the Small Animal Clinic, Faculty of Veterinary Medicine, Ghent University (= clinical population), between September–December 2020 and December 2021 –January 2022. Informed (written) consent was obtained from the dogs’ owners. Sample doubles were removed and breed information, sex, age at the time of collection, and the specialty department through which the dogs were admitted were based on the clinical information provided. Genomic DNA was isolated from the blood samples as previously described [30] and subsequently used for genotyping.

Furthermore, multidrug sensitivity genotyping results obtained from routine genetic testing at the Laboratory of Animal Genetics (Department of Veterinary and Biosciences at the Faculty of Veterinary Medicine in Ghent University) for independent breeders, owners, and veterinarians were gathered (= genetic testing population).

### Genotyping

The standard method used to genotype dogs for the *ABCB1-1Δ* variant was qPCR with dual-labeled probes, while PCR followed by Sanger sequencing was used for lower-quality samples. All genotyping information for both techniques (primer sequences, probe sequences, amplicon length, and details on PCR/sequencing mixes and programs) is provided in the S1 File. The forward primer was used to perform sequencing.

### Statistics

The central aim in this study was to have a sufficient sample size to calculate the risks of symptoms as small as something you encounter in 1 in 10 000 patients (i.e. 0.01%). While population estimates differ, previously published studies found allelic frequencies between 0% and 56% [24]. Comparing several studies, a population allelic frequency estimate of 20% was found. As several (but not all) prevalence estimates from earlier studies were based on genetic testing populations, we wanted to ensure that even when the prevalence was far lower (i.e. 5–10 times as low), we would still be able to detect the allele with a high probability. As such, based on the study of Gregorius (1980), a sample size of 265 was found to be sufficient to find the *ABCB1* variant even if it would occur at a frequency of 1/5^th^ to 1/10^th^ of the earlier reported frequencies with a probability of 99% [31]. In terms of “risk to develop symptoms” calculations, this sample size corresponds to risks as small as 0.003% (i.e. 3 in 100 000 patients), which is even more rare than what we minimally aimed for (i.e. 0.01%) and as such sufficient.

In both populations, the overall and breed-specific frequency of the deletion (del%) was calculated. Using Hardy-Weinberg equilibrium (HWE), the expected proportion of homozygous variant (del/del) and heterozygous (wt/del) dogs in the clinical population was estimated. A 95% confidence interval (CI) for both populations’ del% was calculated, as described by Lewis and Mellersh (2019) [32].

## Results

### Clinical population

In total, 288 samples were gathered and two sample doubles were removed, leaving a test population of 286 samples. The cohort consisted of a very diverse group of 90 different breeds and mixed breeds. While mixed breeds (Canis vulgaris in Table 1.) formed the largest group, German shepherds, Labrador retrievers, golden retrievers, and chihuahuas completed the top five. A complete overview of all breeds, the number of collected samples per breed, and their del% can be found in Table 1 under Clinic. The average and median age of the dogs was 7 years (minimum: 2 months; maximum: 15 years 7 months). The sex was distributed evenly (147 males, 138 females, one unknown). The samples came from 10 different clinical specializations, with the top three being internal medicine (39%), emergency care (26%), and neurology (10%). All information regarding breed, sex, age, and specialty are displayed in the S1 Table. Only one sample, a Shetland sheepdog, was genotyped heterozygous (wt/del) for the *ABCB1-1Δ* variant, while all other samples were homozygous for the wildtype allele (wt/wt), resulting in a del% of 0.2% (95% CI = [0, 0.52%]). Assuming HWE, the expected percentage of homozygous variant (del/del) and wt/del dogs in the clinical population is 0.0003% and 0.35%, respectively.

**Table 1:** List of sampled breeds.

Breed	Clinic		Laboratory		Breed	Clinic		Laboratory	
	n	del%	n	del%		n	del%	n	del%
Afghan hound	-	-	2	0%	Great Münsterländer	1	0%	-	-
Alaskan malamute	2	0%	-	-	Greyhound	1	0%	-	-
American cocker spaniel	3	0%	1	0%	Griffon Bruxellois	1	0%	-	-
American Staffordshire terrier	7	0%	-	-	Husky	3	0%	2	0%
Australian kelpie	1	0%	3	0%	Irish setter	2	0%	-	-
**Australian shepherd**	**3**	**0%**	**120**	**32%**	Irish terrier	1	0%	-	-
Barbet	1	0%	-	-	Italian greyhound	1	0%	1	0%
Basset hound	1	0%	-	-	Jack Russell terrier	7	0%	-	-
Beagle	6	0%	1	0%	Kooikerhondje	1	0%	-	-
**Bearded collie**	**-**	**-**	**5**	**0%**	**Labradoodle**	**3**	**0%**	**-**	**-**
Beauceron	3	0%	-	-	Labrador retriever	12	0%	-	-
Belgian Groenendaeler	1	0%	-	-	Leonberger	2	0%	-	-
Belgian Malinois	9	0%	1	0%	Maltese dog	8	0%	-	-
Belgian Tervuren	1	0%	-	-	Manchester terrier	1	0%	-	-
Bernese mountain dog	9	0%	-	-	Miniature American shepherd	-	-	2	0%
Bichon frisé	3	0%	-	-	**Miniature Australian shepherd**	**-**	**-**	**1**	**0%**
Boerenfox	-	-	1	0%	Norfolk terrier	1	0%	-	-
Boerboel	1	0%	-	-	Nova Scotia duck tolling retriever	1	0%	3	0%
Bolonka zwetna	1	0%	-	-	**Old English sheepdog**	**-**	**-**	**6**	**0.33%**
Bordeaux dog	1	0%	-	-	Papillon	2	0%	-	-
**Border collie**	**9**	**0%**	**174**	**0.6%**	Pembroke Welsh corgi	1	0%	-	-
Bouvier des Ardennes	1	0%	-	-	Petit basset griffon Vendéen	1	0%	-	-
Bouvier des Flandres	3	0%	-	-	Podenco	-	-	3	0%
Boxer	2	0%	-	-	Pomeranian	5	0%	-	-
Bracco Italiano	1	0%	-	-	Poodle (medium)	1	0%	-	-
Briard	1	0%	2	0%	Poodle (toy)	1	0%	-	-
Cane corso Italiano	1	0%	-	-	Portugese water dog	1	0%	-	-
**Canis vulgaris**	**32**	**0%**	**19**	**3%**	Pug	-	-	1	0%
Cavalier King Charles spaniel	7	0%	-	-	Rhodesian ridgeback	4	0%	-	-
Chesapeake Bay retriever	1	0%	-	-	Rottweiler	1	0%	-	-
Chihuahua	10	0%	-	-	Saarloos wolf dog	1	0%	-	-
Chinese crested dog	1	0%	-	-	Saint Bernard	1	0%	-	-
Chow chow	1	0%	-	-	Schapendoes	1	0%	1	0%
**Collie**	**-**	**-**	**89**	**58%**	Shar-Pei	2	0%	-	-
Dachshund	7	0%	-	-	**Shetland sheepdog**	**4**	**12.5%**	**66**	**0.31%**
Dobermann pinscher	5	0%	-	-	Shiba Inu	1	0%	-	-
English bulldog	2	0%	3	0%	Shih Tzu	2	0%	-	-
**English cocker spaniel**	**6**	**0%**	**3**	**0%**	Small Münsterländer	1	0%	-	-
English springer spaniel	1	0%	-	-	Spanish water dog	1	0%	1	
Estrela mountain dog	1	0%	-	-	Spitz	2	0%	-	-
Flatcoated retriever	1	0%	-	-	Stabyhoun	1	0%	-	-
Fox terrier	1	0%	-	-	Staffordshire bull terrier	1	0%	1	0%
French bulldog	7	0%	-	-	Unknown	1	0%	13	0.09%
Galgo Espagñol	3	0%	-	-	Vizsla	3	0%	-	-
German pinscher	1	0%	-	-	Weimaraner	3	0%	1	0%
**German shepherd**	**16**	**0%**	**3**	**0%**	West Highland white terrier	1	0%	-	-
German shorthaired pointer	1	0%	-	-	**Whippet**	**5**	**0%**	**3**	**0%**
Giant schnauzer	1	0%	-	-	**White Swiss shepherd dog**	**1**	**0%**	**67**	**0.22%**
Golden retriever	11	0%	-	-	Yorkshire terrier	3	0%	-	-
Great Dane	1	0	-	-					

(n) The number of samples per breed and (del%) variant allele frequency found for the clinical testing population (Clinic) and the genetic testing population (Genetic). Breeds in which the *ABCB1-1Δ* variant has already been described are indicated in bold. ^a^ Canis vulgaris indicates mixed breeds.

### Genetic testing population

By performing routine genotyping for independent breeders, owners, and veterinarians, *ABCB1-1Δ* genotypes were gathered for 599 dogs. The cohort consisted of 31 breeds and the border collie, Australian shepherd, collie, white Swiss shepherd dog, and Shetland sheepdog formed the largest groups. Except for the border collie, the top 5 breeds are also those in which del/del dogs were found. Only 45 samples (7.5%) were derived from breeds in which the variant allele was absent, while 554 samples (92.5%) came from (mixed) breeds in which the variant allele was detected. An overview of all breeds, the number of collected samples per breed, and their del% are displayed in Table 1 under Genetic. Among the collie, Australian shepherd, Shetland sheepdog, and white Swiss shepherd breed, both del/del and wt/del dogs were found (del% of 58%, 32%, 31%, and 22%, respectively). One dog of an unspecified breed (unknown) also tested del/del. Furthermore, wt/del dogs were detected amongst the Old English sheepdog, border collie, and border collie x collie mixed breeds. The variant was not found in 30 other (cross)breeds. The overall del% of this genetic test population was 21.6% (95% CI = [19.29%, 23.95%]).

## Discussion

While reported first in the collie, multidrug sensitivity caused by the *ABCB1-1Δ* variant has been described in a wide variety of dog breeds: collies and collie-like dogs but also seemingly unrelated breeds [15, 19, 20, 26] and cross-bred dogs [21, 22]. Interestingly, the presence of this variant could be traced back to the collie lineage for the longhaired whippet, silken windhound [15], and white Swiss shepherd dog [18], who all share the *ABCB1-1Δ* variant identical by descent (IBD) with herding breeds from the collie lineage. Though the German shepherd dog could not be traced back in this manner, the variant was mainly found in white dogs or dogs with a white (grand)parent. This link to a white coat and the fact that the white Swiss shepherd (for which IBD was established [18]) originated from the white German shepherd dog indicates the variant has the same origin as all other breeds [24]. However, to the authors’ knowledge, there is no known IBD link between the collie lineage and the cocker spaniel, labradoodle, and several crossbreeds in which the *ABCB1-1Δ* variant has also been reported [20–22]. Furthermore, independent laboratories have reported the presence of the variant in more breeds seemingly unrelated to collies, like the black mouth cur, Carolina dog, and chinook [33]. It should be noted that the *ABCB1-1Δ* variant has only been reported in one labradoodle and one cocker spaniel [20] and while some laboratories report prevalences, they do not always mention the number of dogs tested [33]. As large-scale *ABCB1-1Δ* related studies on these “collie-unrelated” breeds are lacking, variant allele frequencies remain unknown. Furthermore, it cannot be ruled out entirely that crossbreeds and purebred dogs are in actuality mixed-breeds with a collie lineage. Still, this does demonstrate that the variant can sometimes occur in dogs unexpectedly.

Since its discovery, it has been generally advised to genotype breeds at risk for the *ABCB1-1Δ* variant before treating them with risk drugs [9, 27]. As medical professionals, veterinarians regularly administer and prescribe medication to their patients, including drugs that might lead to toxicosis in susceptible dogs. Since *ABCB1-1Δ* genotyping is commercially available in many countries, we expected that veterinarians often request a DNA test for multidrug sensitivity before treating breeds at risk with any risk drug. However, it was recently shown that 55% of veterinarians in a university setting never requested any DNA test [29]. While it is possible that DNA tests were already performed in a subset of patients, especially in a university setting where many of the patients are referrals, this would only bias this 55% result if every patient of those individual veterinarians that needed a DNA test would already have had a result. While DNA tests performed earlier would thus not change the number of veterinarians answering “never”, it might have lowered the frequency of requested DNA tests of the veterinarians that do request DNA tests. Therefore, eventhough Bogaerts et al. (2021) did not specifically investigate multidrug sensitivity testing, the number of veterinarians testing for multidrug sensitivity is thus low.

The low usage of DNA tests indicated by this cross-sectional study and the fact that disease-causing variants can occur unexpectedly in breeds previously not at risk due to (unknown) crossbreeding raised the question of whether there was a great risk to not testing dogs for multidrug sensitivity in a clinical setting. Eventhough Bogaerts et al. (2021) did not specifically investigate multidrug sensitivity and it is possible that testing for this disorder was already previously performed on the dogs in our clinical population (by the breeder or primary veterinarian), the study does point out a general low usage of DNA tests in a university setting.

Instead of genotyping dogs and breeds at risk, it might be that veterinarians adopted a different approach, i.e. lowering the treatment dosage to prevent toxic reactions against risk drugs. However, the recommended dosage for several treatments, such as treatment of generalized demodicosis with ivermectin [5], antidiarrheal treatment with loperamide [6], sedation with acepromazine [7], and lymphoma treatment with vincristine, vinblastine, or doxorubicin [8] were all shown to cause toxicity in multidrug sensitive dogs. Using lower doses of P-gp substrate chemotherapeutic drugs for the treatment of at-risk breeds increases the chance of shorter remission duration [34]. Overall, it is clear that lowering the dosage might reduce treatment efficacy.

Even when low dosages can be employed in risk breeds, the sometimes unexpected and unexplained presence of the *ABCB1-1Δ* variant in breeds outside the collie lineage leaves the question of whether the variant segregates in still unknown breeds. In this case, no preventive measures regarding dosage would be taken, and thus, never testing for the variant as a veterinarian could come with great risks. Therefore, this study estimated the prevalence of the variant in a clinical setting.

As prevalences can vary geographically, a first step was to investigate whether the genetic testing population in Belgium differs from the one found in the literature. Routine genotyping of the *ABCB1-1Δ* variant led to a total del% of 21.6% (95% CI: [19.29%, 23.95%]) in a genetic testing population. High breed-specific Vts%, similar to the ones found in this study, have been reported for collies, Australian shepherds, Shetland sheepdogs, and white Swiss shepherd dogs in studies from all over the world [15–17, 19–28]. These results indicate the Belgian dog population is indeed comparable to what can be found in literature regarding multidrug sensitivity. Notably, 92% of the genetic testing population samples came from breeds in which the variant has been reported before in the literature and 89% of the samples came from breeds in which the variant was found here. It thus seems at-risk breeds often get identified, either by breeders, dog owners, or veterinarians.

The next step was the identification of the *ABCB1-1Δ* variant in a clinical population. Aiming to provide an unbiased estimate, residue EDTA blood samples were collected at random. Out of 286 samples, only one was heterozygous, while all other samples did not carry the variant. As such, a del% of 0.2% (95% CI: [0, 0.52%]) was found for the clinical population. The heterozygous sample belonged to a Shetland sheepdog, one of the already-established breeds at risk for multidrug sensitivity with a del% between 7 and 30%, as shown by several population studies [15–17, 20, 23, 24, 26]. While the sample size is too low to correctly estimate the del% in specific breeds (for many breeds, no or few dogs were included), this was not the main goal of this study. However, the results do provide a rough estimate of the del% in a clinical setting, which is apparently low.

A first observation is that the del% is roughly 100 times higher in the genetic test population compared to the clinical population (21.6% and 0.2%, respectively). As the clinical and genetic testing population originate from the same geographical location and because the del% in the genetic testing population is in the same order of magnitude as what is generally reported, the low prevalence in the clinical population does not seem to be a consequence of a deviation due to the geographical location of the dogs. The difference is thus likely a consequence of various other reasons. It is generally reported that prevalence estimates based on populations presented for genetic testing might be (upward) biased. A direct comparison of the breed-specific prevalence (to accurately quantify this bias) was not possible here due to the generally low numbers of samples in individual breeds. However, this comparison is also not the goal of this study. As the aim is to assess the risk of not genotyping, the overall probability to be presented with multidrug-sensitive dogs (del/del and sometimes wt/del dogs) in a clinical population is important. Here, the HWE calculations based on a del% of 0.2% predicted only 0.0003% of the dogs in the clinical population will be del/del and 0.35% will be wt/del for the *ABCB1-1Δ* variant (0.35% combined). Therefore, the risk of encountering a multidrug-sensitive dog is very low.

This result has to be interpreted with caution of course. Firstly, it depends on the profile of the patients that are presented. Some practices might see more patients of breeds at risk than others. In our clinical sample, 26% of the sampled dogs are from breeds in which the *ABCB1-1Δ* variant has already been reported. A veterinary practice with a relatively higher proportion of those breeds at risk might increase the probability of encountering dogs prone to multidrug sensitivity. However, we expect no bias caused by the samples coming from a university setting, since dogs do not get referred to a secondary practice based on their multidrug sensitivity genotype. Furthermore, as patients in a university clinic are typically referred by primary practitioners, the samples in this study come from dogs covering a large geographical surface (Belgium and the Netherlands).

Importantly, the probability of encountering adverse effects is not identical to the probability that a dog homozygous for the *ABCB1-1Δ* variant is presented. While adverse effects to e.g. ivermectin are rarely seen in heterozygous dogs, they have been noted in some wt/del dogs treated for generalized demodicosis with 300 μg/kg, a dose at the lower end of the scale for mange treatment and tolerated by most (but not all) *ABCB1-1Δ* free dogs [10]. Regarding chemotherapeutics, a preliminary study showed del/del or wt/del dogs are more likely to develop hematologic toxicity after treatment with vincristine than wt/wt dogs [11]. Therefore, we also provided a combined estimate of wt/del and del/del dogs. Additionally, adverse effects are only seen when dogs actually receive risk drugs. To obtain the probability of observing adverse effects, the probability that a “genetically at risk” dog is presented to a veterinarian should be multiplied by the probability a disease occurs for which treatment with risk drugs is necessary (as genotype and administration of drugs are independent events). To give a rough risk estimate of veterinarians facing adverse effects in practice, two treatment examples are provided.

Taking into account that adverse reactions against ivermectin only arise at high treatment doses, mange treatment in a multidrug sensitive dog poses a first good practical example as a daily administration of 300–600 μg/kg is recommended [35]. Acute neurotoxicity occurs in del/del dogs at doses >100 μg/kg [9], while only some wt/del dogs show subchronic neurotoxicity at doses >300 μg/kg [10] and some heterozygotes have also been shown to tolerate doses >600 μg/kg [9]. Importantly, subchronic signs of neurotoxicity cannot only be attributed to the *ABCB1-1Δ* variant, as they are also seen in wt/wt dogs [13]. A recent study in the UK estimated the prevalence of generalized demodicosis as low as 0.46% for juvenile-onset and 0.05% for adult-onset demodicosis. Furthermore, the study reported a breed predisposition in the British bulldog, Staffordshire bull terrier, Chinese shar-pei, dogue de Bordeaux, pug, French bulldog, and boxer, all breeds not predisposed to multidrug sensitivity [36]. Even assuming the worst case scenario, i.e. a combined del/del and wt/del frequency of 0.35%, multiplied with a prevalence of 0.51% demodicosis (= combined prevalence of juvenile and adult-onset demodicosis), only implies a probability of 0.002%. Taking all this into account, the risk of a veterinarian being confronted with a dog showing adverse reactions to ivermectin because of the *ABCB1-1Δ* variant is even lower than 0.35%. Moreover, several alternative treatments which are safe to use in multidrug-sensitive dogs are available [35], rendering the use of ivermectin at such high doses redundant.

Though ivermectin is used more regularly in primary veterinary medicine, chemotherapeutics are used more frequently in secondary veterinary medicine. A risk assessment similar to ivermectin can be performed for the use of vincristine and doxorubicin in canine lymphoma treatment. Canine lymphoma has an estimated annual incidence rate of 13–114 cases per 100000 dogs. Chemotherapy is the therapy of choice and the multi-agent (so-called CHOP) protocols, which include both doxorubicin and vincristine, are currently preferred as they result in the highest response rates and longest response duration [37]. It has been shown that recommended dosages of vincristine and doxorubicin are likely to cause hematologic toxicity in dogs with the *ABCB1-1Δ* variant, both in homozygous or heterozygous form [8, 11]. Among the dog breeds with a significant predisposition to lymphoma [37–39], only the German shepherd and border collie have been reported to carry the multidrug sensitivity variant, and only at a very low frequency [19, 21, 24, 27, 28]. Even though adverse reactions are seen in del/del and wt/del dogs at standard dosages (in contrast to ivermectin treatment of mange), taking into account the clinical population’s del/del and wt/del percentages and the breed predisposition for lymphoma, the risk of encountering adverse reactions to vincristine and/or doxorubicin because of multidrug sensitivity is still a factor five smaller compared to the previous example (0.0004% = 0.35% x 114/100000).

Based on these examples, the overall probability of encountering adverse effects seems to be low, even though genetic testing is not standardly performed. However, a relatively limited risk does not imply that the general recommendations are not valid. As mentioned before, the breed profile can differ between veterinary practices, as well as the diseases and thus the likelihood of administering risk drugs. Furthermore, even if rare in a clinical setting, intoxications resulting from the administration of risk drugs to multidrug sensitive dogs can be life-threatening. As such, we do recommend the careful use of known risk drugs and genetic testing of dogs from breeds at risk.

As a final remark, we chose to evaluate only the extensively validated *ABCB1-1Δ* variant here. In 2010, an ivermectin-sensitive border collie was described to lack this frameshift variant. Though the authors discovered a variant that might be related to ivermectin intolerance in this dog [40], the pathogenicity was not proven and the variant has not been identified in other dogs. As such, the variant was not examined in this study.

## Conclusion

Owing to the high allelic frequency usually reported by genetic laboratories, we were surprised by the low allelic frequency found in the clinical dog population. While the risk of overestimating allelic frequency based on commercial tests is generally mentioned, the results obtained here quantify that this difference can be up to 100 times greater. With an allelic frequency of 0.2% in the clinical population, the overall risk of not genotyping for multidrug sensitivity seems to be limited. Moreover, the one heterozygous dog was a Shetland sheepdog, a breed well known for its predisposition to multidrug sensitivity. As such, the current recommendations to prioritize genotyping efforts for at-risk breeds [9] before treatment with risk drugs at potentially toxic doses, seem to sufficiently limit the risk of drug toxicity.

## Supporting information

S1 File Primer, probe, PCR, qPCR, and sequencing information for *ABCB1-1Δ* genotyping. (DOCX)

S1 Table Sample information clinical population. Sample number (n°), breed, sex, age (in years), and specialization is displayed. The specialization is based on the specialty department the dogs were admitted in the Small Animal Clinic. Specialization/departments are: (BLO) Blood donation. (CAR) Cardiology. (DER) Dermatology. (EME) Emergency care. (IMA) Medical imaging. (INT) internal medicine. (NEU) Neurology. (NUT) Nutrition. (ORT) Orthopedics. (SUR) Surgery.
(DOCX)

## References

[1] Campbell WC, Benz GW. Ivermectin: a review of efficacy and safety. Journal of Veterinary Pharmacology and Therapeutics. 1984;7:1–16. doi: 10.1111/j.1365-2885.1984.tb00872.x 6368862

[2] Campbell WC, Fisher MH, Stapley EO, Albers-Schönberg G, Jacob TA. Ivermectin: A Potent New Antiparasitic Agent. Science. 1983 Aug 26;221:823–8. doi: 10.1126/science.6308762 6308762

[3] Mealey KL, Bentjen SA, Gay JM, Cantor GH. Ivermectin sensitivity in collies is associated with a deletion mutation of the mdr1 gene. Pharmacogenetics. 2001;11:727–33. doi: 10.1097/00008571-200111000-00012 11692082

[4] Fromm MF. Importance of P-glycoprotein at blood-tissue barriers. Trends in Pharmacological Sciences. 2004;25(8):423–9. doi: 10.1016/j.tips.2004.06.002 15276711

[5] Merola VM, Eubig PA. Toxicology of Avermectins and Milbemycins (Macrocyclic Lactones) and the Role of P-Glycoprotein in Dogs and Cats. Veterinary Clinics of North America: Small Animal Practice. 2018 Nov;48:991–1012. 30139545 10.1016/j.cvsm.2018.07.002

[6] Sartor LL, Bentjen SA, Trepanier L, Mealey KL. Loperamide Toxicity in a Collie with the MDR1 Mutation Associated with Ivermectin Sensitivity. Journal of Veterinary Internal Medicine. 2004 Jan;18:117–8. doi: 10.1892/0891-6640(2004)18&lt;117:ltiacw&gt;2.0.co;2 14765742

[7] Deshpande D, Hill KE, Mealey KL, Chambers JP, Gieseg MA. The Effect of the Canine ABCB1 ‐1Δ Mutation on Sedation after Intravenous Administration of Acepromazine. Journal of Veterinary Internal Medicine. 2016 Mar 29;30:636–41. doi: 10.1111/jvim.13827 26822006 PMC4913601

[8] Mealey KL, Northrup NC, Bentjen SA. Increased toxicity of P-glycoprotein-substrate chemotherapeutic agents in a dog with the MDR1 deletion mutation associated with ivermectin sensitivity. Journal of the American Veterinary Medical Association. 2003;223(10):1453–5. doi: 10.2460/javma.2003.223.1453 14627096

[9] Geyer J, Janko C. Treatment of MDR1 Mutant Dogs with Macrocyclic Lactones. Current Pharmaceutical Biotechnology. 2012;13:969–86. doi: 10.2174/138920112800399301 22039792 PMC3419875

[10] Mealey KL. Adverse drug reactions in herding-breed dogs: The role of P-glycoprotein. Compendium on Continuing Education for the Practicing Veterinarian. 2006;28:23–33.

[11] Mealey KL, Fidel J, Gay JM, Impellizeri JA, Clifford CA, Bergman PJ. ABCB1-1Δ Polymorphism Can Predict Hematologic Toxicity in Dogs Treated with Vincristine. Journal of Veterinary Internal Medicine. 2008 Jul;22:996–1000. 18537875 10.1111/j.1939-1676.2008.0122.x

[12] Mackin AJ, Riggs C, Beatty T, Mealey K, Boothe D, Archer T. Excessive Cyclosporine-Associated Immunosuppression in a Dog Heterozygous for the MDR1 (ABCB1-1Δ) Mutation. Journal of the American Animal Hospital Association. 2020;56:190. doi: 10.5326/JAAHA-MS-7004 32182109

[13] Bissonnette S, Paradis M, Daneau I, Silversides DW. The ABCB1-1Δ mutation is not responsible for subchronic neurotoxicity seen in dogs of non-collie breeds following macrocyclic lactone treatment for generalized demodicosis. Veterinary Dermatology. 2009 Feb;20:60–6. 19152588 10.1111/j.1365-3164.2008.00731.x

[14] Nelson OL, Carsten E, Bentjen SA, Mealey KL. Ivermectin toxicity in an Australian Shepherd dog with the MDR1 mutation associated with ivermectin sensitivity in Collies. Journal of veterinary internal medicine. 2003;17:354–6. 12774979

[15] Neff MW, Robertson KR, Wong AK, Safra N, Broman KW, Slatkin M, et al. Breed distribution and history of canine mdr1-1Δ, a pharmacogenetic mutation that marks the emergence of breeds from the collie lineage. Proceedings of the National Academy of Sciences. 2004 Aug 10;101(32):11725–30. 10.1073/pnas.0402374101 PMC511012 15289602

[16] Geyer J, Döring B, Godoy JR, Leidolf R, Moritz A, Petzinger E. Frequency of the nt230 (del4) MDR1 mutation in Collies and related dog breeds in Germany. Journal of Veterinary Pharmacology and Therapeutics. 2005;28:545–51. doi: 10.1111/j.1365-2885.2005.00692.x 16343287

[17] Marelli SP, Polli M, Frattini S, Cortellari M, Rizzi R, Crepaldi P. Genotypic and allelic frequencies of MDR1 gene in dogs in Italy. Veterinary Record Open. 2020;7:e000375. doi: 10.1136/vetreco-2019-000375 32617164 PMC7319724

[18] Geyer J, Klintzsch S, Meerkamp K, Wöhlke A, Distl O, Moritz A, et al. Detection of the nt230(del4) MDR1 mutation in White Swiss Shepherd dogs: Case reports of doramectin toxicosis, breed predisposition, and microsatellite analysis. Journal of Veterinary Pharmacology and Therapeutics. 2007;30(5):482–5. doi: 10.1111/j.1365-2885.2007.00885.x 17803743

[19] Pires A da R, Gerardi D, Tisotti T de M, Serpa PB da S, Natalini CC. First report of nt230(del4) mutation in the MDR1 gene in German Shepherds in Southern Brazil. Ciência Rural. 2021;51(10):e20200690.

[20] Soussa R, Woodward A, Marty M, Cannon C. Breed is associated with the ABCB1‐1Δ mutation in Australian dogs. Australian Veterinary Journal. 2020 Mar 19;98:79–83. doi: 10.1111/avj.12896 31743433

[21] Dekel Y, Machluf Y, Stoler A, Aderet A, Baumel D, Kellerman E, et al. Frequency of canine nt230(del4) MDR1 mutation in prone pure breeds, their crosses and mongrels in Israel—insights from a worldwide comparative perspective. BMC Veterinary Research. 2017 Dec 13;13:333. doi: 10.1186/s12917-017-1251-9 29132368 PMC5683241

[22] Donner J, Anderson H, Davison S, Hughes AM, Bouirmane J, Lindqvist J, et al. Frequency and distribution of 152 genetic disease variants in over 100,000 mixed breed and purebred dogs. Leeb T, editor. PLOS Genetics. 2018 Apr 30;14(4):e1007361. doi: 10.1371/journal.pgen.1007361 29708978 PMC5945203

[23] Gramer I, Leidolf R, Döring B, Klintzsch S, Krämer EM, Yalcin E, et al. Breed distribution of the nt230(del4) MDR1 mutation in dogs. Veterinary Journal. 2011;189:67–71. doi: 10.1016/j.tvjl.2010.06.012 20655253

[24] Mealey KL, Meurs KM. Breed distribution of the ABCB1-1Δ (multidrug sensitivity) polymorphism among dogs undergoing ABCB1 genotyping. Journal of the American Veterinary Medical Association. 2008;233:921–4. 18795852 10.2460/javma.233.6.921

[25] Mealey KL, Munyard KA, Bentjen SA. Frequency of the mutant MDR1 allele associated with multidrug sensitivity in a sample of herding breed dogs living in Australia. Veterinary Parasitology. 2005;131:193–6. doi: 10.1016/j.vetpar.2005.05.004 15975717

[26] Firdova Z, Turnova E, Bielikova M, Turna J, Dudas A. The prevalence of ABCB1: c.227_230delATAG mutation in affected dog breeds from European countries. Research in Veterinary Science. 2016;106:89–92. doi: 10.1016/j.rvsc.2016.03.016 27234542

[27] Erkens T, Daminet S, Rogiers C, Gommeren K, Lampo E, Vander Donckt D, et al. Presence of the ABCB1 (MDR1) deletion mutation causing ivermectin hypersensitivity in certain dog breeds in Belgium. Vlaams Diergeneeskundig Tijdschrift. 2009;78:256–60.

[28] Beckers E, Van Poucke M, Ronsyn L, Peelman L. Frequency estimation of disease-causing mutations in the Belgian population of some dog breeds—Part 1: shepherds. Vlaams Diergeneeskundig Tijdschrift. 2016;85:175–84.

[29] Bogaerts E, den Boer E, Peelman L, Van Nieuwerburgh F, Fieten H, Saunders JH, et al. Veterinarians’ Competence in Applying Basic Genetic Principles and Daily Implementation of Clinical Genetics: A Study in a University Environment. Journal of Veterinary Medical Education. 2021;e20210029. doi: 10.3138/jvme-2020-0029 36472562

[30] Van Poucke M, Vandesompele J, Mattheeuws M, Van Zeveren A, Peelman LJ. A dual fluorescent multiprobe assay for prion protein genotyping in sheep. BMC Infectious Diseases. 2005 Dec 15;5:13. doi: 10.1186/1471-2334-5-13 15769289 PMC1274271

[31] Gregorius HR. The Probability of Losing an Allele When Diploid Genotypes are Sampled. Biometrics. 1980 Dec;36(4):643–52. 7248433

[32] Lewis TW, Mellersh CS. Changes in mutation frequency of eight Mendelian inherited disorders in eight pedigree dog populations following introduction of a commercial DNA test. Loor JJ, editor. PLOS ONE. 2019 Jan 16;14(1):e0209864. doi: 10.1371/journal.pone.0209864 30650096 PMC6334900

[33] Laboratory WVCP. Breeds affected by the MDR1 mutation (frequency %) [Internet]. Washington State University College of Veterinary Medicine. 2022 [cited 2022 Apr 15]. Available from: http://vcpl.vetmed.wsu.edu/affected-breeds

[34] Mealey KL. Therapeutic implications of the MDR-1 gene. Journal of Veterinary Pharmacology and Therapeutics. 2004 Oct;27:257–64. doi: 10.1111/j.1365-2885.2004.00607.x 15500562

[35] Mueller RS, Rosenkrantz W, Bensignor E, Karaś‐Tęcza J, Paterson T, Shipstone MA. Diagnosis and treatment of demodicosis in dogs and cats. Veterinary Dermatology. 2020 Feb 19;31:4–e2. 10.1111/vde.12806 31957202

[36] O’Neill DG, Turgoose E, Church DB, Brodbelt DC, Hendricks A. Juvenile‐onset and adult‐onset demodicosis in dogs in the UK: prevalence and breed associations. Journal of Small Animal Practice. 2020 Jan 4;61:32–41. doi: 10.1111/jsap.13067 31584708 PMC7003809

[37] Zandvliet M. Canine lymphoma: a review. Veterinary Quarterly. 2016 Apr 2;36(2):76–104. doi: 10.1080/01652176.2016.1152633 26953614

[38] Villamil JA, Henry CJ, Hahn AW, Bryan JN, Tyler JW, Caldwell CW. Hormonal and Sex Impact on the Epidemiology of Canine Lymphoma. Journal of Cancer Epidemiology. 2009;2009:1–7. doi: 10.1155/2009/591753 20445802 PMC2859020

[39] Van Rooyen LJ, Hooijberg E, Reyers F. Breed prevalence of canine lymphoma in South Africa. Journal of the South African Veterinary Association. 2018 Mar 8;89:1–11. doi: 10.4102/jsava.v89i0.1530 29781671 PMC6138093

[40] Han JI, Son HW, Park SC, Na KJ. Novel insertion mutation of ABCB1 gene in an ivermectin-sensitive Border Collie. Journal of Veterinary Science. 2010;11(4):341–4. doi: 10.4142/jvs.2010.11.4.341 21113104 PMC2998746

