# Supplementary material for: The prevalence of the ABCB1-1Δ variant in a clinical veterinary setting: The risk of not genotyping
Source: PLoS One. 2022 Aug 29;17(8):e0273706. doi: 10.1371/journal.pone.0273706 (PMC9423603; doi:10.1371/journal.pone.0273706)
Supplement: S1 File — (DOCX) [file pone.0273706.s001.docx]

**S1 File. Primer, probe, PCR, qPCR, and sequencing information for *ABCB1-1Δ* genotyping.**

**Table 1.** Sequences of the primers and probes used for *ABCB1-1Δ* genotyping, and amplicon length. F and R indicate forward and reverse primers, and Wt and Vt indicate the probes binding to the wildtype and variant strand, respectively.

| **Primers** | **Probes** | **Amplicon length (bp)** |
| --- | --- | --- |
| F: 5’-GATAGGTTGTATATGTTGGTG-3’ | Wt: HEX-TGCAAAGCTATCTGTCATGTTTC-BHQ1 | 180 |
| R: 5’-CTAAGATCAGTGCCACAA-3’ | Vt: TR-TTGCAAAGCTGTCATGTTTCC-BHQ2 |  |

**Table 2.** PCR/qPCR/sequencing mixes and programs used for *ABCB1-1Δ* genotyping.

| **PCR mix:**  5.7 µl H_2_O (Thermo Fisher Scientific, Waltham, MA, USA)  1.0 µl 10x Key buffer (VWR International, Radnor, PA, USA)  1.0 µl Primers (5 µM each) (Integrated DNA Technologies, Coralville, IA, USA)  0.2 µl dNTPs (10 mM each) (VWR International)  0.1 µl TEMPase Hotstart DNA polymerase (5 U/µl) (VWR International)  2.0 µl Template  10.0 µl Total volume | **PCR program:**  14'30" - 95°C  00'30" - 95°C ]  00'30" - 63°C ] x 35  01'00" - 72°C ]  04'00" - 72°C  Hold - 15°C |
| --- | --- |
| **qPCR mix:**  5.5 µl H_2_O  1.0 µl 10x Key buffer  0.4 µl Primers (5 µM each)  0.4 µl Wt probe (10 µM) (Integrated DNA Technologies)  0.4 µl Vt probe (10 µM) (Integrated DNA Technologies)  0.2 µl dNTPs (10 mM each)  0.1 µl TEMPase Hotstart DNA polymerase (5 U/µl)  2.0 µl Template  10.0 µl Total volume | **qPCR program:**  14'30" - 95°C  00'30" - 95°C ]  00'30" - 63°C ] x 35  01'00" - 72°C ]  04'00" - 72°C  Hold - 15°C |
| **Sequencing mix:**  3.0 µl H_2_O  2.0 µl 5x SEQ-buffer (Thermo Fisher Scientific, Waltham, MA, USA)  1.5 µl Sequencing primer (2 µM)  1.0 µl GC-rich solution (Roche Diagnostics, Mannheim)  0.5 µl RR-mix (Thermo Fisher Scientific, Waltham, MA, USA)  2.0 µl Template  10.0 µl Total volume | **Sequencing program:**  2'00" - 95°C  0'20" - 95°C ]  0'10" - 60°C ] 30x  4'00" - 65°C ]  Hold - 15°C |
