# Supplementary material for: The prevalence of the ABCB1-1Δ variant in a clinical veterinary setting: The risk of not genotyping
Source: PLoS One. 2022 Aug 29;17(8):e0273706. doi: 10.1371/journal.pone.0273706 (PMC9423603; doi:10.1371/journal.pone.0273706)
Supplement: S1 Table — Sample number (n°), breed, sex, age (in years), and specialization is displayed. The specialization is based on the specialty department the dogs were admitted in the Small Animal Clinic. Specialization/departments are: (BLO) Blood donation. (CAR) Cardiology. (DER) Dermatology. (EME) Emergency care. (IMA) Medical imaging. (INT) internal medicine. (NEU) Neurology. (NUT) Nutrition. (ORT) Orthopedics. (SUR) Surgery. (DOCX) [file pone.0273706.s002.docx]

**S2 Table. Sample information clinical population.**

| **Sample n°** | **Breed** | **Sex** | **Genotype** | **Age** | **Specialization** |
| --- | --- | --- | --- | --- | --- |
| 1 | Golden retriever | m | wt/wt | 9.3 | INT |
| 2 | Dobermann pinscher | m | wt/wt | 9.5 | INT |
| 3 | Golden retriever | f | wt/wt | 11.6 | INT |
| 4 | Poodle (toy) | m | wt/wt | 12.2 | DER |
| 5 | Labrador retriever | f | wt/wt | 4.4 | EME |
| 6 | Border collie | f | wt/wt | 4.0 | NEU |
| 7 | Belgian Malinois | m | wt/wt | 6.1 | NEU |
| 8 | Papillon | f | wt/wt | 2.3 | INT |
| 9 | Labrador retriever | m | wt/wt | 9.3 | INT |
| 10 | Shetland sheepdog | m | wt/wt | 9.4 | ORT |
| 11 | Chow chow | m | wt/wt | 5.4 | BLO |
| 12 | Poodle (medium) | m | wt/wt | 13.0 | INT |
| 13 | American Staffordshire terrier | f | wt/wt | 4.6 | INT |
| 14 | Chihuahua | m | wt/wt | 9.3 | INT |
| 15 | Whippet | m | wt/wt | 7.5 | INT |
| 16 | Golden retriever | m | wt/wt | 11.4 | SUR |
| 17 | Stabyhoun | f | wt/wt | 4.4 | NEU |
| 18 | English cocker spaniel | m | wt/wt | 5.4 | ORT |
| 19 | Canis vulgaris | m | wt/wt | 12.8 | NEU |
| 20 | Jack Russell terrier | m | wt/wt | 13.3 | CAR |
| 21 | Golden retriever | f | wt/wt | 3.4 | ORT |
| 22 | Whippet | m | wt/wt | 9.5 | DER |
| 23 | Saint Bernard | m | wt/wt | 5.6 | CAR |
| 24 | Bernese mountain dog | f | wt/wt | 6.2 | NEU |
| 25 | Cavalier King Charles spaniel | m | wt/wt | 10.6 | CAR |
| 26 | Shih Tzu | m | wt/wt | 4.8 | EME |
| 27 | Yorkshire terrier | m | wt/wt | 12.4 | ORT |
| 28 | German shepherd | f | wt/wt | 6.1 | DER |
| 29 | Saarloos wolf dog | m | wt/wt | 1.7 | INT |
| 30 | Golden retriever | f | wt/wt | 7.0 | SUR |
| 31 | American Staffordshire terrier | m | wt/wt | 3.7 | BLO |
| 32 | Boxer | m | wt/wt | 10.2 | INT |
| 33 | Maltese dog | m | wt/wt | 8.2 | INT |
| 34 | Golden retriever | m | wt/wt | 11.8 | INT |
| 35 | Labrador retriever | m | wt/wt | 10.8 | EME |
| 36 | Dachshund | f | wt/wt | 7.0 | NEU |
| 37 | Border collie | m | wt/wt | 3.7 | INT |
| 38 | Whippet | m | wt/wt | 9.0 | INT |
| 39 | Bernese mountain dog | f | wt/wt | 7.2 | INT |
| 40 | Maltese dog | m | wt/wt | 9.8 | ORT |
| 41 | German shepherd | m | wt/wt | 3.3 | ORT |
| 42 | Golden retriever | f | wt/wt | 8.0 | INT |
| 43 | Maltese dog | f | wt/wt | 9.9 | EME |
| 44 | Leonberger | f | wt/wt | 2.2 | INT |
| 45 | Labrador retriever | m | wt/wt | 8.9 | INT |
| 46 | Canis vulgaris | f | wt/wt | 5.9 | INT |
| 47 | Labrador retriever | m | wt/wt | 4.3 | NEU |
| 48 | Chihuahua | m | wt/wt | 9.4 | INT |
| 49 | American Staffordshire terrier | f | wt/wt | 10.3 | ORT |
| 50 | Canis vulgaris | m | wt/wt | 5.8 | NEU |
| 51 | American Staffordshire terrier | m | wt/wt | 7.7 | EME |
| 52 | Rottweiler | f | wt/wt | 0.2 | EME |
| 53 | Beagle | f | wt/wt | 7.0 | SUR |
| 54 | Great Dane | m | wt/wt | 2.7 | INT |
| 55 | Leonberger | f | wt/wt | 7.3 | BLO |
| 56 | Canis vulgaris | f | wt/wt | 13.0 | INT |
| 57 | Chihuahua | f | wt/wt | 0.5 | EME |
| 58 | Labradoodle | m | wt/wt | 0.8 | EME |
| 59 | Chihuahua | m | wt/wt | 11.4 | INT |
| 60 | Bernese mountain dog | m | wt/wt | 0.3 | EME |
| 61 | Golden retriever | m | wt/wt | 3.1 | EME |
| 62 | French bulldog | m | wt/wt | 11.5 | EME |
| 63 | Canis vulgaris | m | wt/wt | 6.0 | INT |
| 64 | Petit basset griffon Vendéen | f | wt/wt | 9.2 | INT |
| 65 | Shetland sheepdog | m | wt/del | 3.8 | INT |
| 66 | Maltese dog | f | wt/wt | 11.9 | EME |
| 67 | German shepherd | m | wt/wt | 3.8 | INT |
| 68 | Bracco Italiano | m | wt/wt | 11.2 | EME |
| 69 | German shepherd | f | wt/wt | 2.2 | INT |
| 70 | Weimaraner | m | wt/wt | 6.4 | INT |
| 71 | Belgian Malinois | m | wt/wt | 8.8 | ORT |
| 72 | Papillon | f | wt/wt | 7.8 | INT |
| 73 | Cane corso Italiano | f | wt/wt | 7.9 | INT |
| 74 | Labrador retriever | f | wt/wt | 12.2 | INT |
| 75 | French bulldog | m | wt/wt | 5.1 | NEU |
| 76 | Beagle | f | wt/wt | 7.4 | INT |
| 77 | Great Münsterländer | f | wt/wt | 9.4 | EME |
| 78 | Labrador retriever | f | wt/wt | 3.9 | INT |
| 79 | Canis vulgaris | f | wt/wt | 0.4 | INT |
| 80 | Canis vulgaris | m | wt/wt | 2.9 | INT |
| 81 | Shar-Pei | m | wt/wt | 8.5 | INT |
| 82 | Canis vulgaris | f | wt/wt | 3.3 | INT |
| 83 | Canis vulgaris | f | wt/wt | 7.8 | EME |
| 84 | Border collie | m | wt/wt | 5.4 | EME |
| 85 | Pomeranian | f | wt/wt | 4.5 | NEU |
| 86 | White shepherd dog | m | wt/wt | 1.5 | IMA |
| 87 | Bernese mountain dog | m | wt/wt | 6.9 | NEU |
| 88 | Alaskan malamute | f | wt/wt | 5.7 | ORT |
| 89 | Bernese mountain dog | m | wt/wt | 1.7 | BLO |
| 90 | Shetland sheepdog | m | wt/wt | 0.7 | CAR |
| 91 | Spaanse water dog | f | wt/wt | 8.3 | DER |
| 92 | Bichon frisé | m | wt/wt | 12.7 | INT |
| 93 | Vizsla | f | wt/wt | 6.0 | INT |
| 94 | Chihuahua | f | wt/wt | 13.3 | NEU |
| 95 | Belgian Groenendaeler | f | wt/wt | 3.1 | NEU |
| 96 | Canis vulgaris | m | wt/wt | 2.1 | NEU |
| 97 | Dachshund | f | wt/wt | 4.2 | EME |
| 98 | Chihuahua | f | wt/wt | 0.8 | EME |
| 99 | Maltese dog | f | wt/wt | 3.8 | NEU |
| 100 | Canis vulgaris | f | wt/wt | 13.5 | INT |
| 101 | Dachshund | m | wt/wt | 4.6 | NEU |
| 102 | Belgian Malinois | f | wt/wt | 7.5 | INT |
| 103 | Beagle | f | wt/wt | 10.5 | SUR |
| 104 | German shepherd | f | wt/wt | 5.4 | EME |
| 105 | Jack Russell terrier | m | wt/wt | 9.0 | EME |
| 106 | Basset hound | f | wt/wt | 10.6 | ORT |
| 107 | German shorthaired pointer | f | wt/wt | 8.1 | ORT |
| 108 | Maltese dog | f | wt/wt | 5.5 | INT |
| 109 | Irish setter | f | wt/wt | 1.4 | EME |
| 110 | West Highland white terrier | f | wt/wt | 13.4 | EME |
| 111 | Bouvier des Ardennes | m | wt/wt | 8.8 | INT |
| 112 | Dobermann pinscher | m | wt/wt | 7.6 | INT |
| 113 | American Staffordshire terrier | m | wt/wt | 10.0 | SUR |
| 114 | Dachshund | m | wt/wt | 6.8 | EME |
| 115 | English bulldog | f | wt/wt | 6.8 | SUR |
| 116 | Bernese mountain dog | f | wt/wt | 0.8 | EME |
| 117 | Rhodesian ridgeback | m | wt/wt | 12.8 | ORT |
| 118 | Kooikerhondje | m | wt/wt | 5.1 | INT |
| 119 | English bulldog | f | wt/wt | 2.3 | EME |
| 120 | Chihuahua | f | wt/wt | 12.2 | INT |
| 121 | Whippet | f | wt/wt | 11.7 | EME |
| 122 | Maltese dog | m | wt/wt | 2.8 | INT |
| 123 | Cavalier King Charles spaniel | m | wt/wt | 5.3 | CAR |
| 124 | Golden retriever | f | wt/wt | 5.0 | NEU |
| 125 | Pomeranian | f | wt/wt | 1.0 | INT |
| 126 | Bordeaux dog | m | wt/wt | 1.2 | EME |
| 127 | Bouvier des Flandres | m | wt/wt | 10.3 | EME |
| 128 | Chihuahua | f | wt/wt | 8.9 | EME |
| 129 | English cocker spaniel | m | wt/wt | 11.4 | ORT |
| 130 | German shepherd | m | wt/wt | 1.2 | EME |
| 131 | Shar-Pei | m | wt/wt | 10.1 | EME |
| 132 | Pembroke Welsh Corgi | m | wt/wt | 6.8 | EME |
| 133 | Rhodesian ridgeback | m | wt/wt | 0.3 | EME |
| 134 | Cavalier King Charles spaniel | f | wt/wt | 9.1 | EME |
| 135 | Rhodesian ridgeback | m | wt/wt | 9.6 | ORT |
| 136 | Canis vulgaris | f | wt/wt | 8.5 | INT |
| 137 | Galgo Espagñol | f | wt/wt | 2.2 | CAR |
| 138 | Canis vulgaris | m | wt/wt | 0.7 | EME |
| 139 | Canis vulgaris | m | wt/wt | 10.4 | INT |
| 140 | Onbekend | ? | wt/wt | ? | ? |
| 141 | Irish Terrier | f | wt/wt | 6.0 | EME |
| 142 | Labradoodle | f | wt/wt | 2.6 | INT |
| 143 | Labradoodle | f | wt/wt | 1.2 | INT |
| 144 | Vizsla | f | wt/wt | 7.0 | INT |
| 145 | Cavalier King Charles | f | wt/wt | 9.1 | INT |
| 146 | Labrador retriever | m | wt/wt | 9.2 | INT |
| 147 | Pomerian | m | wt/wt | 3.5 | EME |
| 148 | Beagle | f | wt/wt | 4.8 | NEU |
| 149 | Canis vulgaris | f | wt/wt | 0.2 | EME |
| 150 | Canis vulgaris | m | wt/wt | 8.5 | EME |
| 151 | German Pinscher | m | wt/wt | 12.3 | INT |
| 152 | Border collie | m | wt/wt | 14.5 | EME |
| 153 | Flatcoated Retriever | f | wt/wt | 8.4 | INT |
| 154 | Belgian Malinois | m | wt/wt | 9.8 | EME |
| 155 | Norfolk Terrier | f | wt/wt | 2.0 | INT |
| 156 | American Staffordshire terrier | f | wt/wt | 10.5 | EME |
| 157 | Manchester Terrier | f | wt/wt | 7.5 | EME |
| 158 | German shepherd | f | wt/wt | 8.3 | EME |
| 159 | Galgo Espagñol | f | wt/wt | 11.8 | INT |
| 160 | Beauceron | m | wt/wt | 9.4 | EME |
| 161 | Belgian Malinois | f | wt/wt | 5.0 | EME |
| 162 | Weimaraner | f | wt/wt | 6.8 | BLO |
| 163 | Yorkshire Terrier | f | wt/wt | 14.7 | INT |
| 164 | Chihuahua | m | wt/wt | 11.3 | CAR |
| 165 | American Staffordshire terrier | m | wt/wt | 4.3 | INT |
| 166 | Dobermann pinscher | m | wt/wt | 8.8 | INT |
| 167 | Labrador retriever | f | wt/wt | 3.3 | BLO |
| 168 | Australian shepherd | m | wt/wt | 9.5 | INT |
| 169 | Bouvier des Flandres | m | wt/wt | 10.7 | ORT |
| 170 | Canis vulgaris | m | wt/wt | 10.6 | INT |
| 171 | Border collie | m | wt/wt | 4.4 | NUT |
| 172 | Canis vulgaris | f | wt/wt | 3.3 | EME |
| 173 | Canis vulgaris | f | wt/wt | 7.8 | EME |
| 174 | Greyhound | f | wt/wt | 9.6 | NEU |
| 175 | Canis vulgaris | m | wt/wt | 3.9 | EME |
| 176 | Briard | m | wt/wt | 7.7 | EME |
| 177 | Border collie | f | wt/wt | 5.8 | NUT |
| 178 | Staffordshire Bull Terrier | m | wt/wt | 11.8 | NEU |
| 179 | English Cocker Spaniel | f | wt/wt | 4.2 | EME |
| 180 | Maltese dog | m | wt/wt | 6.3 | INT |
| 181 | Canis vulgaris | m | wt/wt | 10.8 | INT |
| 182 | American cocker spaniel | m | wt/wt | 7.5 | INT |
| 183 | English Cocker Spaniel | m | wt/wt | 6.9 | INT |
| 184 | German shepherd | f | wt/wt | 5.3 | BLO |
| 185 | Canis vulgaris | m | wt/wt | 6.9 | INT |
| 186 | Golden retriever | f | wt/wt | 7.9 | SUR |
| 187 | Chesapeake Bay Retriever | m | wt/wt | 2.9 | BLO |
| 188 | Canis vulgaris | m | wt/wt | 10.4 | SUR |
| 189 | Alaskan malamute | f | wt/wt | 7.1 | EME |
| 190 | Cavalier King Charles | f | wt/wt | 5.3 | INT |
| 191 | American cocker spaniel | f | wt/wt | 4.9 | NEU |
| 192 | Dachshund | f | wt/wt | 11.3 | INT |
| 193 | Dobermann pinscher | m | wt/wt | 3.5 | INT |
| 194 | French Bulldog | f | wt/wt | 4.3 | EME |
| 195 | Australian shepherd | m | wt/wt | 1.9 | EME |
| 196 | Shih Tzu | m | wt/wt | 7.3 | EME |
| 197 | German shepherd | f | wt/wt | 10.2 | INT |
| 198 | French Bulldog | m | wt/wt | 6.0 | INT |
| 199 | Yorkshire Terrier | m | wt/wt | 8.9 | EME |
| 200 | Belgian Malinois | f | wt/wt | 2.7 | EME |
| 201 | Canis vulgaris | f | wt/wt | 13.6 | EME |
| 202 | Irish Setter | f | wt/wt | 9.9 | INT |
| 203 | Australian kelpie | m | wt/wt | 2.6 | EME |
| 204 | Beagle | m | wt/wt | 12.3 | EME |
| 205 | French Bulldog | m | wt/wt | 1.9 | NEU |
| 206 | Border collie | f | wt/wt | 2.8 | INT |
| 207 | Jack Russell terrier | m | wt/wt | 11.8 | INT |
| 208 | Spitz | m | wt/wt | 10.9 | ORT |
| 209 | Border collie | f | wt/wt | 9.0 | ORT |
| 210 | Estrela Mountain Dog | m | wt/wt | 5.4 | INT |
| 211 | Australian shepherd | f | wt/wt | 4.7 | INT |
| 212 | Pomerian | f | wt/wt | 6.6 | INT |
| 213 | Shiba Inu | m | wt/wt | 7.0 | INT |
| 214 | German shepherd | m | wt/wt | 10.5 | EME |
| 215 | Labrador retriever | m | wt/wt | 5.8 | INT |
| 216 | Fox Terrier | m | wt/wt | 3.8 | SUR |
| 217 | Canis vulgaris | m | wt/wt | 9.8 | INT |
| 218 | English Cocker Spaniel | f | wt/wt | 8.6 | NEU |
| 219 | Bouvier des Flandres | f | wt/wt | 10.5 | EME |
| 220 | Jack Russell terrier | f | wt/wt | 11.9 | INT |
| 221 | Jack Russell terrier | m | wt/wt | 14.2 | INT |
| 222 | Canis vulgaris | m | wt/wt | 7.3 | EME |
| 223 | Husky | m | wt/wt | 5.5 | CAR |
| 224 | Labrador retriever | m | wt/wt | 9.4 | ORT |
| 225 | German shepherd | f | wt/wt | 1.0 | BLO |
| 226 | Small Munsterlander | m | wt/wt | 5.9 | INT |
| 227 | Shetland sheepdog | m | wt/wt | 0.8 | INT |
| 228 | Golden retriever | m | wt/wt | 2.8 | INT |
| 229 | French Bulldog | m | wt/wt | 3.8 | EME |
| 230 | Barbet | m | wt/wt | 10.9 | CAR |
| 231 | German shepherd | f | wt/wt | 11.5 | EME |
| 232 | Border collie | f | wt/wt | 0.3 | CAR |
| 233 | Bernese mountain dog | m | wt/wt | 6.2 | ORT |
| 234 | Boerboel | f | wt/wt | 6.3 | SUR |
| 235 | Dachshund | f | wt/wt | 6.3 | NEU |
| 236 | Vizsla | f | wt/wt | 11.7 | EME |
| 237 | Chihuahua | f | wt/wt | 2.1 | NEU |
| 238 | Boxer | f | wt/wt | 4.7 | EME |
| 239 | Beagle | f | wt/wt | 10.3 | SUR |
| 240 | Bernese mountain dog | m | wt/wt | 7.8 | ORT |
| 241 | Canis vulgaris | f | wt/wt | 5.6 | SUR |
| 242 | Beauceron | m | wt/wt | 7.0 | INT |
| 243 | Riesenschnauzer | f | wt/wt | 5.3 | INT |
| 244 | Canis vulgaris | m | wt/wt | 13.4 | INT |
| 245 | Canis vulgaris | m | wt/wt | 5.4 | INT |
| 246 | German shepherd | m | wt/wt | 6.9 | SUR |
| 247 | Chinese crested dog | f | wt/wt | 11.0 | INT |
| 248 | Griffon Bruxellois | m | wt/wt | 12.2 | CAR |
| 249 | Canis vulgaris | f | wt/wt | 5.0 | INT |
| 250 | Belgian Tervuren | m | wt/wt | 6.0 | NEU |
| 251 | Jack Russell terrier | f | wt/wt | 5.2 | NEU |
| 252 | Bernese mountain dog | f | wt/wt | 4.2 | NEU |
| 253 | Bolonka zwetna | f | wt/wt | 1.3 | INT |
| 254 | Spitz | f | wt/wt | 11.2 | SUR |
| 255 | Cavalier King Charles | m | wt/wt | 9.9 | CAR |
| 256 | Galgo Espagñol | m | wt/wt | 6.8 | SUR |
| 257 | Nova Scotia Duck Tolling retriever | m | wt/wt | 6.5 | INT |
| 258 | Italian greyhound | m | wt/wt | 10.5 | INT |
| 259 | Canis vulgaris | m | wt/wt | 7.3 | EME |
| 260 | German shepherd | f | wt/wt | 0.6 | ORT |
| 261 | Portugese water dog | f | wt/wt | 4.4 | NEU |
| 262 | Canis vulgaris | f | wt/wt | 5.2 | NUT |
| 263 | French bulldog | f | wt/wt | 2.8 | EME |
| 264 | Cavalier King Charles spaniel | f | wt/wt | 8.0 | EME |
| 265 | Pomeranian | f | wt/wt | 9.0 | EME |
| 266 | German shepherd | m | wt/wt | 5.8 | INT |
| 267 | Belgian Malinois | f | wt/wt | 7.0 | BLO |
| 268 | Whippet | f | wt/wt | 8.6 | INT |
| 269 | Jack Russell terrier | f | wt/wt | 0.8 | NEU |
| 270 | English springer spaniel | m | wt/wt | 9.3 | EME |
| 271 | English cocker spaniel | m | wt/wt | 6.7 | INT |
| 272 | Schapendoes | m | wt/wt | 2.0 | EME |
| 273 | Husky | f | wt/wt | 2.5 | INT |
| 274 | American cocker spaniel | f | wt/wt | 15.6 | EME |
| 275 | Rhodesian ridgeback | m | wt/wt | 6.5 | INT |
| 276 | Bichon frisé | m | wt/wt | 13.1 | EME |
| 277 | Belgian Malinois | m | wt/wt | 8.2 | INT |
| 278 | Dobermann pinscher | m | wt/wt | 13.4 | INT |
| 279 | Beauceron | m | wt/wt | 7.0 | INT |
| 280 | Husky | f | wt/wt | 11.5 | INT |
| 281 | Weimaraner | f | wt/wt | 11.7 | SUR |
| 282 | Belgian Malinois | f | wt/wt | 7.5 | BLO |
| 283 | Labrador retriever | f | wt/wt | 9.3 | INT |
| 284 | Bichon frisé | f | wt/wt | 13.9 | INT |
| 285 | Dachshund | f | wt/wt | 12.1 | SUR |
| 286 | German shepherd | f | wt/wt | 7.3 | INT |

Sample number (n°), breed, sex, genotype, age (in years), and specialization is displayed. The specialization is based on the specialty department the dogs were admitted in the Small Animal Clinic. Specialization/departments are: (BLO) Blood donation. (CAR) Cardiology. (DER) Dermatology. (EME) Emergency care. (IMA) Medical imaging. (INT) internal medicine. (NEU) Neurology. (NUT) Nutrition. (ORT) Orthopedics. (SUR) Surgery.
